# Generating novel tennis racket shape concepts using a theoretical morphospace

**DOI:** 10.1371/journal.pone.0310155

**Published:** 2024-09-12

**Authors:** Robyn A. Grant, Vincent Bonhomme, Tom Allen

**Affiliations:** 1 Faculty of Science and Engineering, Manchester Metropolitan University, Manchester, United Kingdom; 2 Athéna, Lacamp, Roquedur, France; 3 ISEM, University Montpellier, Montpellier, France; University of Liverpool, UNITED KINGDOM OF GREAT BRITAIN AND NORTHERN IRELAND

## Abstract

Statistical shape analysis, or morphometrics, is a technique commonly used in evolutionary biology to summarise a population of samples. Theoretical morphometrics extends the current population of samples into a theoretical space. Using the lawn tennis racket as an example, this paper showcases the potential of morphometrics as a tool for inspiring design concepts for novel sporting goods. It showcases how morphometrics can be applied to summarise the shape of a sample of rackets and applies theoretical morphometrics to systematically present new candidate designs that fall outside of the inputted existing, racket population. The input population was 514 tennis rackets dating back to the origins of the game. The shape analysis was performed on “front-on” silhouette images of the rackets using the R Package Momocs. The outline shape of each racket was reconstructed using the elliptical Fourier transform curve fitting technique. A principal component analysis performed on the reconstructed outlines showed that >90% of the variance in the shape of the rackets was captured by the first two principal components. An evenly spaced grid of theoretical racket shape outlines was then created in a principal component 2 vs. principal component 1 “morphospace”. The limits of this space were then expanded to give a theoretical morphospace that extended beyond the range of the first two principal components for the 514 samples. We propose that the shapes located within such a theoretical morphospace could inspire designers and help them to systematically identify candidates for novel products. Specifically, we suggest that experimenting with wide-angled throats and heads with angled sides might be an interesting starting point for exploring future tennis racket design concepts. The novel technique presented here could be used by a sporting goods brand during the ideation phase of product development to schematically summarise current designs and identify candidates for new ones.

## Introduction

The design of sporting goods can influence athlete performance and injury risk. Sports brands engage in research and development to bring new and improved products to their customers. Such improvements in sporting goods are often incremental, with occasional step changes [1–5]. These continual improvements can benefit customers, such as by making a sport easier to learn, which can increase accessibility and enjoyment. New design approaches could help accelerate the development of sporting goods and bring societal benefits, such as increased uptake of sport and exercise and reduced injury risk.

Current design approaches often look to previous designs to inform, or inspire, new concepts, for example by using generative design. Such generative design tools often use genetic models and artificial intelligence (AI) to iteratively create concepts, which are usually constrained to certain design specifications [6,7]. Commonly, these concepts are produced in computer-aided design (CAD) software to give three-dimensional shapes [6]. Image datasets can also be used to train AI models to generate design concepts [8]. However, AI is often considered a “black box”, and although explainable AI is revealing many of the processes behind its decision-making [9–11], a more systematic approach might often be preferable.

Shape grammars offer one such systematic approach to generative design [12–15]. They are of the replacement type of generative design algorithm, which means a part of a shape is replaced by another during a transformation to make a new one. Shape rules determine which part of the shape is to be transformed and how. Repeatedly applying these shape rules generates different shapes. There are various extensions of basic shape grammars, including those that are parametric, which allow shape distortions during transformations. As such, shape grammars can form the basis for parametric design and optimisation techniques [12], which can be used to optimise the shape of sporting goods to meet design specifications [16–18]. However, shape grammars are constrained by user inputs, that are especially needed to specify the shape rules and design specifications. Such a requirement for use inputs can limit objectivity, exploratory flexibility, and design freedom.

Geometric morphometrics is often used in evolutionary biology and palaeontology to summarise the shape of a sample population of natural objects. The technique has also been applied to summarise the shape of manmade objects, including tennis rackets [19] and violins [20]. In evolutionary biology, *theoretical morphospaces* [21] are used to propose all possibilities of a given form, including those that fall outside the sample population. There are two steps to using theoretical morphometrics. The first is to map the existing shapes within a sample population, this can be done using manual points, or elliptical harmonics. Elliptical harmonics uses an equation to capture the outline shape of a silhouette. Simple adjustments to the input variables in the equation can transform the shape of the outlines. Therefore, a representative shape can be created for any point on the morphospace, even without data from that area. The whole area can also be extended into a theoretical zone, which is the second step in the process, which identifies novel shapes above and beyond the current population. Using this technique, we can go beyond the bounds of previous designs and extend into a theoretical space [21], to systematically and efficiently explore candidate design concepts that have not been used before.

The lawn tennis racket makes an ideal test case for studying the history of sporting goods and the exploration of new design approaches. Its design has changed a lot since it was conceived around 150 years ago [1,19,22–24], which has helped make tennis easier to learn and play. The earliest tennis rackets were wooden, with long handles and small asymmetric heads. The asymmetric head was soon phased out, with wooden rackets retained until around the 1970s. A subsequent period of experimentation with various materials led to the modern racket design, which is made of fibre-polymer composites and has a shorter handle and a larger head than its wooden predecessor.

We previously quantified how tennis racket shape had developed over time by applying two-dimensional morphometric analyses to >500 samples dating back to the 1870s [19]. As the data required for this technique is images, it is more efficient than taking manual measurements and facilitates analysis of many samples [19,20]. We showed how innovations in materials, especially composites, allowed designers to overcome the mechanical constraints of wood and make rackets with larger and more oval shaped heads. Indeed, with advances in materials, tennis racket shape is no longer restricted by the properties of wood. Which means that, provided they adhere to governing body regulations (of length and width), designers now have the freedom to produce a diverse range of racket shapes. Despite this design freedom, tennis racket shape has barely changed this century [19].

This paper furthers our previous investigation of tennis racket shape (19) by exploring the application of theoretical morphospaces to generate new design concepts. Using images of >500 tennis rackets spanning >140 years [19], we apply this technique to systematically identify and explore new possible shapes. The techniques presented have broad implications for the design of sporting goods, particularly those for which shape is important, such as bikes [2,3], boards [17,18], projectiles [25], rackets [19] and skis [4,26].

## Materials and methods

### Samples

The dataset of racket photographs used here was collected by Taraborelli et al. [1] and presented in Grant et al. [19]. It contained front-on photographs of 514 tennis rackets from 1874 to 2017. The rackets were from the Wimbledon Lawn Tennis Museum (n = 412), a brand’s headquarters (n = 90), the International Tennis Federation (n = 4) and Manchester Metropolitan University (n = 8). They were photographed from above when laid flat on a white background.

### Shape analysis

**Fig 1:**
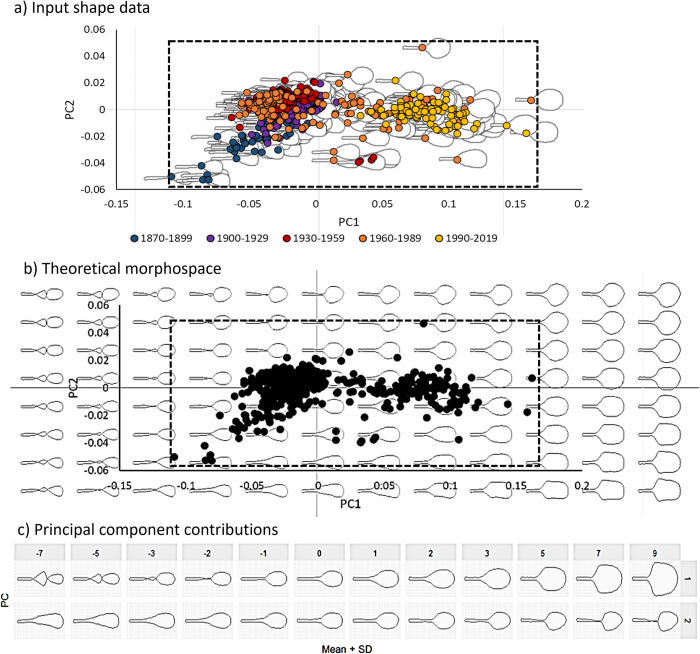
Tennis racket morphospaces based on the two main principal components (PC1 and PC2). Panel a) shows the input shape data of 514 rackets, and panel b) shows the theoretical morphospace, both around the input data (within the dashed line box) and over an extended theoretical range of ± 2 standard deviations. Dashed line box identifies the actual data space in panel a) and b). Panel c) shows the PC contributions of PC1 and PC2 individually, when all other principal components are held at their mean values.

We used the.jpg racket silhouette images from Grant et al. [19], which were extracted from the photographs of Taraborelli et al. [1] (see Fig 1 of [19]). As done before [19], the 514 silhouette images were imported into the R package Momocs [27]. Racket (x, y) outlines were then extracted (with 1,685 ± 211 coordinates per outline) and aligned using a full generalised Procrustes alignment using three landmark points (left and right base handle points and the top point of the head). Outline (x, y) coordinates were then transformed into quantitative variables using elliptical Fourier transforms (see [28]) with 25 harmonics (Fig 1A). The quality of the generated racket outlines was confirmed by eye. As there were 25 harmonics, each with four coefficients, there were 100 quantitative variables for each racket outline. When these variables for all the rackets were entered in a principal component (PC) analysis they gave 100 principal components. The principal component analysis was conducted using the ‘PCA’ command in the statistical software R (R version 4.3.1). All 100 principal components for the 514 input racket shapes are in the Supporting Information of this manuscript.

The first two principal components (PC1 and PC2) for each racket were plotted to show the shape space they occupied, termed *morphospace* (Fig 1A). The first two principal components for this dataset have been described in Grant et al. [19], and readers are referred there for an in-depth summary of the racket metrics associated with them. Briefly, PC1 was correlated to racket head width, head length, mass, frame depth and length, as well as polar and transverse (about a lateral in-plane axis passing through the butt) moments of inertia (MOI). PC2 was not correlated to any of the racket metrics. The morphospace contained an outline for each racket at the position of its PC1 and PC2 values. As these racket outlines were modelled using elliptic harmonics there was an equation capturing the shape of each one. This means that changing the variables in the equation changes the shape of the racket outline. Using the plot_PCA function in Momocs, this process can be used to generate a grid of evenly spaced *theoretical* racket shapes.

We produced a 12 by 8 grid of 96 theoretical racket shapes, plotted across the PC2 vs PC1 morphospace (Fig 1B). These 96 theoretical shapes covered the space occupied by the 514 rackets, plus an extended range of ±2 standard deviations in both PC1 and PC2. Extending the range of PC1 and PC2 values allowed examination of racket shapes associated with the unoccupied *theoretical morphospace*. The PC contributions were examined further using the *PCcontrib* function in Momocs with mean ± standard deviation extensions of PC1 and PC2 individually (-7, -5, -3, -2, -1, 1, 2, 3, 5, 7, 9), with the other PCs held at their mean value (Fig 1C). From the *theoretical* morphospace, we visually identified common racket shape features and will define these further in the results section. To investigate the effect of input silhouette samples on the *theoretical morphospace* output, and to align the work more closely to current products, we repeated the analysis on a subset of 84 modern rackets (post-1990). We again extended the range to ±2 standard deviations of PC1 and PC2 (for this subset of modern rackets) and presented a grid of 11 by 9 rackets.

## Results

### Full racket sample

PC1 (86%) and PC2 (5%) together captured >90% of the variation in racket shape and the morphospace plot can be seen in Fig 1. The large variance (>90%) predicted by these first two components and the steep decline in percentage variance for sequential components (observed using a scree plot) suggests that they were sufficient for capturing meaningful variation in racket shape [29]. Indeed, PC1 alone was probably sufficient for capturing meaningful variation in racket shape, although at least two components are needed to plot a 2D morphospace. Fig 1B shows the extended morphospace (± 2 standard deviations) of PC1 and PC2 with theoretical racket shapes. Looking solely across the PC1 x-axis, we can see that at high values (on the right side of the theoretical morphospace in Fig 1B, and with high positive standard deviations in Fig 1C), the rackets are larger with open, simple shapes; whereas smaller and more complex rackets with intricate handles can be seen at low PC1 values (on the left side of the theoretical morphospace in Fig 1B, with high negative standard deviations in Fig 1C). On the PC2 y-axis, we can see that at high values, rackets have rounder heads (at the top of the theoretical morphospace in Fig 1B, and with high positive standard deviations in Fig 1C), and have narrow throats and long racket heads at lower values of PC2 (at the bottom of the theoretical morphospace in Fig 1B, and with high negative standard deviations in Fig 1C). Rather than considering the PC1 and PC2 values separately, other patterns can also be seen when looking across the morphospace. For instance, high values of PC1 and PC2 (top right of Fig 1B) are associated with wide-angled heads. At intermediate values of PC1 and low values of PC2 (center bottom of Fig 1B) we also tend to see rackets with narrow-angled throats.

**Fig 2:**
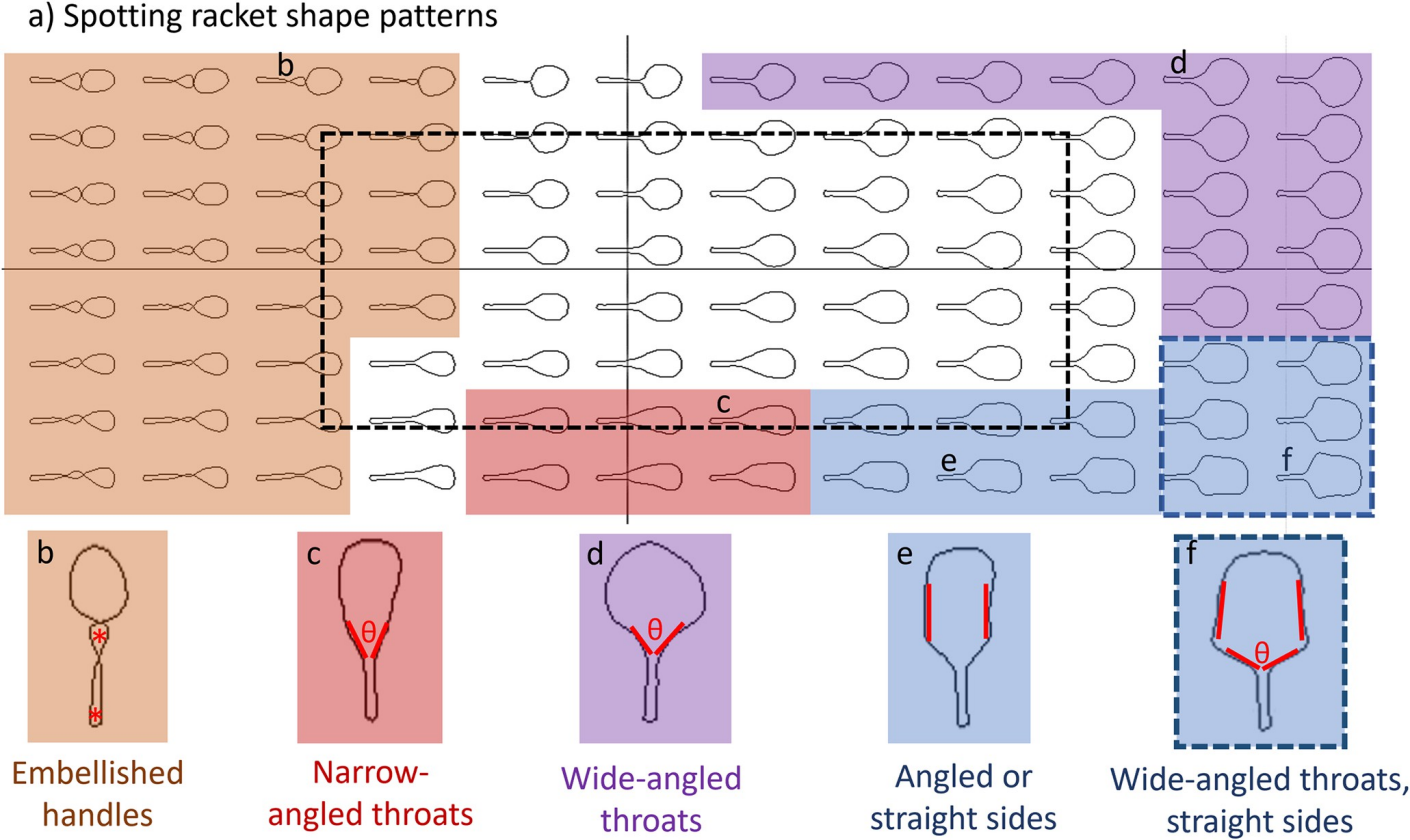
Identifying racket shape patterns from the theoretical morphospace based on the two main principal components (PC1 and PC2). Panel a) shows the theoretical morphospace, both around the input data (within the dashed line box) and over an extended theoretical range of ± 2 standard deviations. Dashed line box identifies the actual data space. Inset panels show main shape features identified by-eye, including embellished handles (orange area and inset panel b), narrow-angled throats (red area and inset c), wide-angled throats (purple area and inset d and f), and angled or straight sides (blue area and inset panels e and f). The dashed blue box indicates an overlap of the two features wide-angled throats and angled or straight sides. The red annotations on the inset panels show how the features are defined, by handle compartmentalisation (*), throat angle (θ) and orientation of the head sides.

Indeed, looking across the whole theoretical morphospace (Fig 1B), we can identify common groups of features in distinct areas of the space (Fig 2A). The low values of PC1 on the left side of the plot all show rackets with complex, embellished handles (in orange in Fig 2A) that almost have figure-of-eight shapes at the lowest PC1 values (Fig 2B). Narrow throat examples can be seen at low values of PC2 and middle values of PC1 (red in Fig 2A and 2C). At the right-hand side of the plot, at high values of PC1, we see rackets with wide-angled throats (in purple and blue Fig 2A and 2D–2F). At low values of PC2, these examples can also have straight or angled sides to the head of the racket (in blue in dashed line box in Fig 2A and 2F). The blue area in Fig 2A (and examples in Fig 2E and 2F) shows racket shape examples with straight or angled sides to the head. The groupings of these features can be defined more formally, as indicated by the red annotations on the inset panels in Fig 2. Embellished handles can be described by handle compartmentalisation, where one or more loops can be observed on the racket handle outline (* in Fig 2B). Throat angle (θ in Fig 2C, 2D and 2F) can be used to define narrow-angled throats at below 40° (Fig 2C) and wide-angled throats at above 90° (Fig 2D and 2F), with input racket samples having throat angles of 45–80°. Instances of angled or straight-sided racket heads can be identified when the racket head outline is not a smooth curve but interrupted by angled sections. These include both straight sides (as in Fig 2E) and angled (Fig 2F) sections of the head.

### Reduced sample analysis on modern rackets

**Fig 3:**
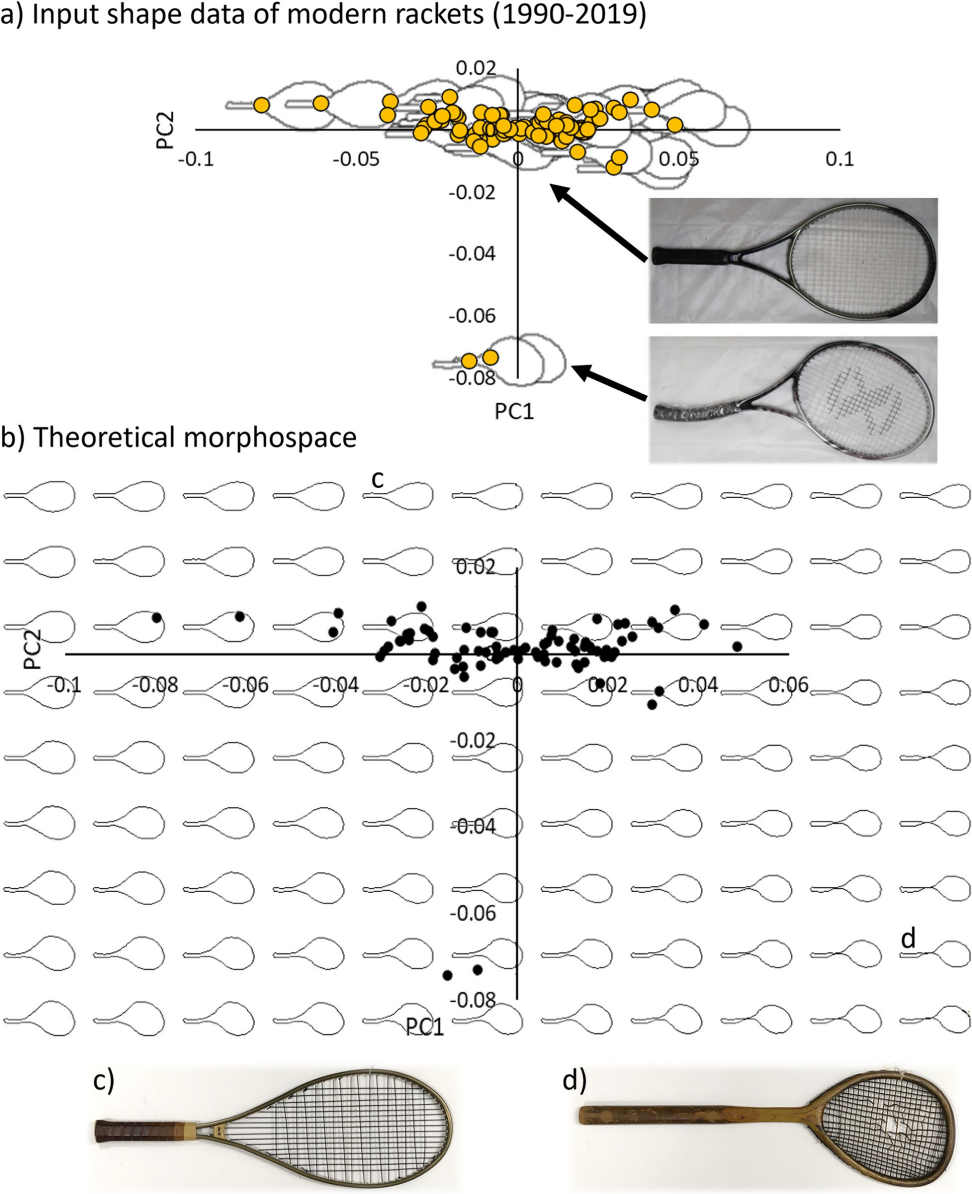
Tennis racket morphospaces based on the two main principal components (PC1 and PC2) of modern rackets (post-1990). Panel a) shows the input shape data of 84 rackets, and panel b) shows the theoretical morphospace, both around the input data (within the dashed line box) and over an extended theoretical range of ± 2 standard deviations. Inset photographs show the two representative racket shapes over the morphospace—a symmetric, oval head (e.g., Wilson, Triad 2.0 Hammer 2003, top) and asymmetric head (e.g., Neoxx, ST 285 2008, bottom). Panels c) and d) show existing, older example rackets that are similar to the theoretical outlines. c) is a Keubler Plus 60 racket from the 1980s and d) is a Lambert racket from 1874.

When the analysis was repeated on a sub-set of modern rackets (n = 84, Fig 3), PC1 (66%) and PC2 (20%) together captured >85% of the variance in racket shape. The corresponding morphospace plot in Fig 3 has been extended by the same range of ± 2 standard deviations as the previous example with all the rackets (Figs 1 and 2). Looking at the input shapes of the rackets, there is a main cluster of those which are of the conventional symmetric design (Fig 3A top cluster of 82 points) and a separately located pair that are asymmetric (Fig 3A, bottom two points, example photographs inset). In the theoretical morphospace, asymmetry (particularly of the handle) seems to be associated with PC2, with more asymmetric rackets at the bottom (negative y-axis part) of the space (Fig 3B), where the two unconventional samples are located. PC1 appears to be associated with head size, with larger racket head sizes on the left hand-side of the plot and smaller ones on the right (Fig 3B). Due to the smaller variation in this sub-set of modern rackets, there is less shape diversity shown within the same ±2 standard deviations theoretical morphospace, than the one in Fig 1B, which was constructed using all the samples. Some areas of the theoretical morphospace (beyond the input data sample) reveals shapes that resemble the older rackets, including those with long, narrow-angled throats (Fig 3C) and those that are asymmetric with relatively small heads (Fig 3D).

## Discussion

We apply here a novel design technique to systematically explore new possible shapes for tennis rackets, with wider implications for the development of sporting goods. By using two-dimensional morphometric shape analyses and constructing a theoretical morphospace (± 2 standard deviations of PC1 and PC2 of 514 racket shapes), we can visualise concepts for new racket shapes. Investigating this space by-eye, we identified candidate features that could be explored more in future designs, including rackets with wide-angled throats (>90°), angled or straight-sided heads, narrow-angled throats (<45°) and embellished handles.

**Fig 4:**
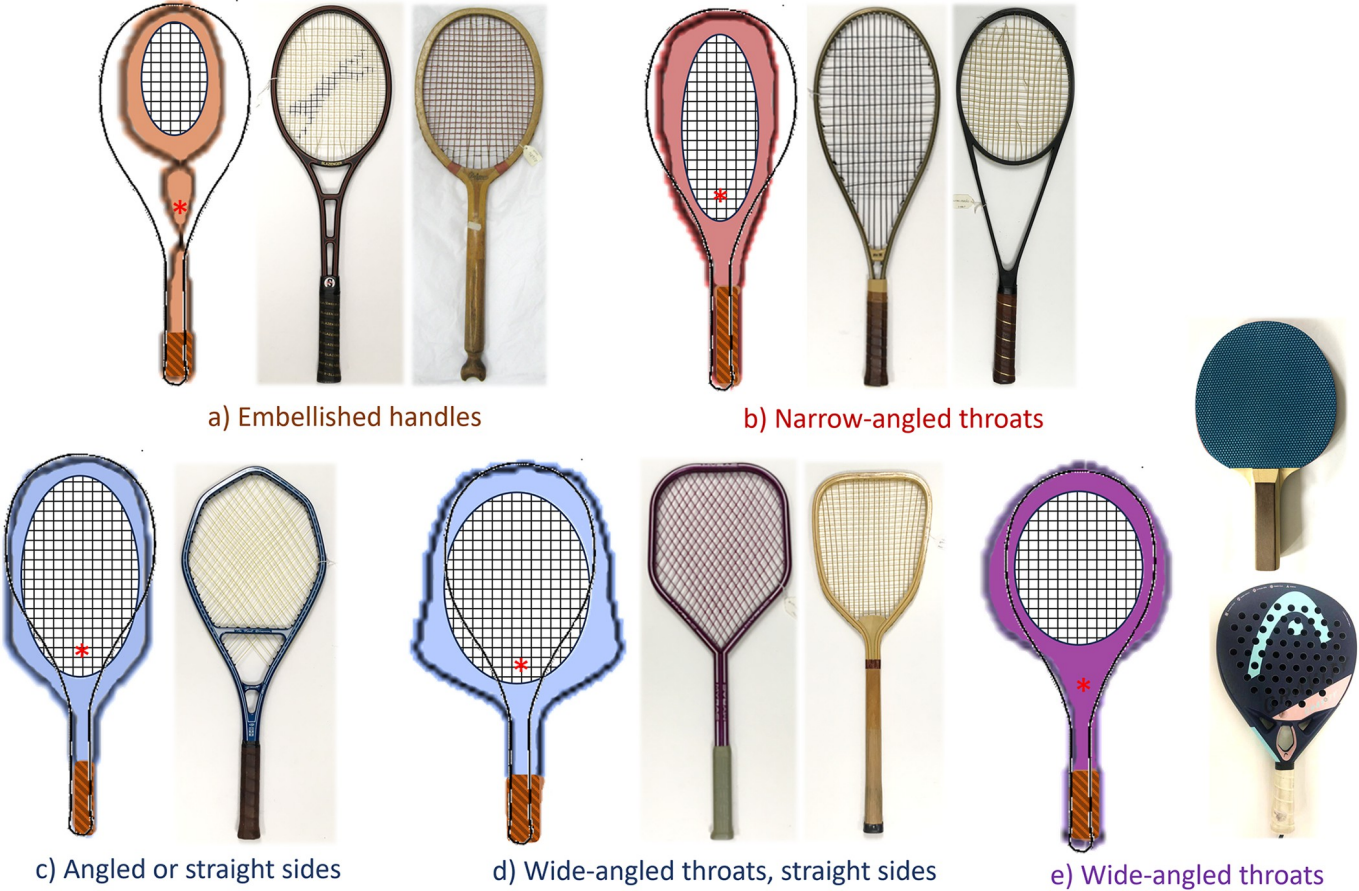
Example rackets with features from the theoretical morphospace in Fig 1B. Including a) Embellished handles: Slazenger Phantom 1974; Defiance 1900; b) Narrow-angled throats: Keubler Plus 60 1980; Silver Arrow 1983; c) Angled or straight sides: Bonny Bergelin Long String 1986; d) Wide-angled throats with straight sides: Inter Business AG, Myrac, 1980; Reproduction racket from 1885; e) wide-angled throats: Table tennis bat; a Padel racket (Head Gravity Motion 2022). A mean composite racket outline is overlaid on each concept racket, taken from Fig 3D of Grant et al. (19). The center of area of each concept image is approximated by a red asterisk (*), as a basic indication of the centre of mass location for that shape.

While the extreme edges of the morphospace (in the extremes of Fig 1B and 1C) might appear to be more theoretical, and unlike typical tennis racket shapes, features of these have appeared before. Fig 4 shows examples of rackets within the dataset with i) embellished handles (Fig 4A), ii) narrow-angled throats (Fig 4B), iii) heads with angled or straight sides (Fig 4C); iv) wide-angled throats with straight sides (Fig 4D), and v) wide-angled throats (Fig 4E). While the shapes of these rackets are not identical to the theoretical ones in the morphospace plot, they do have similar features. For example, the embellished handles become an impractical figure-of-eight shape at extremely low values of PC1 (in orange in Fig 2A). While we have not observed rackets with such handles during our work, compartmented handles have been observed (Fig 4A), and some older wooden rackets had handles with carved features, such as the fish tail in Fig 4A.

One shape feature that was not in our dataset, but was apparent in the theoretical morphospace, furthest from our data points, was rackets with wide-angled throats and heads with curved sides (i.e., in purple in Figs 2 and 4E). While this style of shape is not seen in tennis rackets, it is common in table tennis bats (which have fairly round heads), and also Padel rackets (Fig 4E). This feature may be worth exploring in future tennis racket designs, especially at the extreme of the theoretical morphospace (such as in the high values of PC1 in Fig 1C), where it might even take the form of a spade-shape (♠). A wider head, such as in these shapes with wide-angled throats (including wide-angled throats with straight sides, Fig 4D, and wide-angled throats Fig 4E), may make the racket more resistant to rotation about the longitudinal axis and more ‘stable’ during ‘off-axis’ ball impacts, since racket width is well-correlated to polar MOI [30,31]. The shapes with narrow-angled throats (Fig 4B) are hence likely to have the opposite characteristic—having a low polar MOI and being relatively unstable during play. The difference in width between the head shapes can be clearly seen in Fig 1C. Moving from 0 to 9 standard deviations of PC1 shows a clear increase in head width, which would lead to larger polar moments of inertia.

Having angular, rather than circular heads (Fig 4C and 4D), wide-angled throats (Fig 4D and 4E) and handle embellishments all may cause the centre of mass of the racket to be closer to its butt than in modern examples. For example, a spade-shaped (♠) racket, and some of the examples in Fig 4D, would have less material towards the top of the racket head than around the throat region, which would shift the centre of mass towards the butt. The centre of mass location of a racket is well-correlated to its transverse MOI [30]. A reduction in transverse MOI (from shifting its centre of mass towards the butt) reduces a racket’s resistance to acceleration when swinging and may increase swing speed during serving [32,33] and groundstrokes [34]. Therefore, experimenting with wide-angled throats and heads with angled sides might be an interesting starting point for exploring future tennis racket design concepts.

Indeed, we propose that investigating theoretical morphospaces could provide a new way to systematically identify novel design concepts for sporting goods (such as the examples sketched in Fig 4). The technique has the capacity to be applied to 2- or 3-dimensional forms, and even integrated within computer aided design (CAD) and computer aided engineering (CAE) software. For example, a tennis racket brand could create a theoretical morphospace using existing CAD models of their products to help them visualise the design space they already occupy while systematically identifying new candidate shapes. Geometries of competitor products could also be included in such an analysis to help visualise differences and similarities between them, the overall space occupied by the designs, and even extended to investigate design opportunities. Shortlisting of candidate designs in the theoretical space (such as those in Fig 4) could involve using design specifications as well as quantitative techniques, such as the pareto genetic algorithm [7]. Finite element analysis [35,36] or other analytical techniques [30] could then be adopted to virtually test the candidate racket shapes (e.g., [37–39]), focussing on how they influence inertial properties (such as the centre of mass location and moments of inertia), before prototyping and further assessment. Such integration could streamline design processes, by automatically generating, virtually testing and refining candidate designs based on previous ones.

Composites have allowed engineers to produce diversely shaped rackets [1]. It is worth bearing in mind that different combinations, orientations and amounts of material can be placed around the racket to vary inertial, stiffness and strength properties [40]. Mass can also be added at specific locations of a racket to adjust its inertial properties. This means rackets with the same shape can have very different properties. Indeed, tennis racket development is complex and multifaceted, including aspects of player equipment interaction and preference [41–44] alongside traditional engineering. While the techniques presented here are intended to inspire engineers to explore new racket shapes and concepts, alone they cannot provide information as to whether players and the market will ultimately like and embrace such designs. We, therefore, suggest techniques like those presented here could really only contribute to enhancing the ideation phase of tennis racket development.

The input sample has a large effect on the resulting theoretical morphospace construction. Indeed, modern racket shapes (post-1990) are less diverse than older ones. So, when we used a less diverse sub-set of modern rackets (n = 84), the corresponding candidate shapes in the (± 2 standard deviations) theoretical morphospace varied less (Fig 3B), and mainly only varied in head size and asymmetry (Fig 3A). The shapes in that theoretical morphospace were also clearly influenced by the two unconventional asymmetrical rackets, which were located away from the main cluster and somewhat skewed the results. However, interestingly, extremes of the morphospace (i.e., high values of PC1 and PC2) showed racket features outside of the input sample but reminiscent of the older rackets in our original sample, such as those with long, narrow-angled throats (Fig 4C) and those with small, asymmetric heads (Fig 4D). This may suggest that the theoretical morphospace method can be used to identify past design trends, as well as future possibilities. Indeed, this is often how the technique is used in evolutionary biology, to explore all the possible shapes of a feature that may have occurred in the fossil record [21,45,46]. Aside from sporting goods brands, governing bodies, such as the International Tennis Federation, and museums, such as the Wimbledon Lawn Tennis Museum, could benefit from insights into current and potential shapes for tennis rackets, with implications for regulation, education and player and fan engagement. Future work could explore the application of the techniques presented here to other items of sporting goods, such as bikes [2,3], boards [17,18], projectiles [25] and skis [4,26].

## Supporting information

S1 Data All 100 principal components for the 514 input racket shapes. (CSV)
